# Paraspinal Muscle Health is Related to Fibrogenic, Adipogenic, and Myogenic Gene Expression in Patients with Lumbar Spine Pathology

**DOI:** 10.1186/s12891-022-05572-7

**Published:** 2022-06-24

**Authors:** Brad Anderson, Angel Ordaz, Vinko Zlomislic, R. Todd Allen, Steven R. Garfin, Regula Schuepbach, Mazda Farshad, Simon Schenk, Samuel R. Ward, Bahar Shahidi

**Affiliations:** 1 Department of Orthopaedic Surgery, University of California San Diego, 350 Dickinson Street, Suite 121, Mail Code 8894, San Diego, CA 92103-8894 USA; 2 Department of Orthopaedics, Balgrist University Hospital, University of Zurich, Forchstrasse 340, 8008 Zurich, Switzerland

**Keywords:** Low back pain, Skeletal muscle, Multifidus, Degeneration, Atrophy, Fatty infiltration, Muscle health, Lumbar spine pathology, Surgery

## Abstract

**Background:**

Lumbar spine pathology is a common feature of lower back and/or lower extremity pain and is associated with observable degenerative changes in the lumbar paraspinal muscles that are associated with poor clinical prognosis. Despite the commonly observed phenotype of muscle degeneration in this patient population, its underlying molecular mechanisms are not well understood. The aim of this study was to investigate the relationships between groups of genes within the atrophic, myogenic, fibrogenic, adipogenic, and inflammatory pathways and multifidus muscle health in individuals undergoing surgery for lumbar spine pathology.

**Methods:**

Multifidus muscle biopsies were obtained from patients (*n* = 59) undergoing surgery for lumbar spine pathology to analyze 42 genes from relevant adipogenic/metabolic, atrophic, fibrogenic, inflammatory, and myogenic gene pathways using quantitative polymerase chain reaction. Multifidus muscle morphology was examined preoperatively in these patients at the level and side of biopsy using T2-weighted magnetic resonance imaging to determine whole muscle compartment area, lean muscle area, fat cross-sectional areas, and proportion of fat within the muscle compartment. These measures were used to investigate the relationships between gene expression patterns and muscle size and quality.

**Results:**

Relationships between gene expression and imaging revealed significant associations between decreased expression of adipogenic/metabolic gene (PPARD), increased expression of fibrogenic gene (COL3A1), and lower fat fraction on MRI (*r* = -0.346, *p* = 0.018, and *r* = 0.386, *p* = 0.047 respectively). Decreased expression of myogenic gene (mTOR) was related to greater lean muscle cross-sectional area (*r* = 0.388, *p* = 0.045).

**Conclusion:**

Fibrogenic and adipogenic/metabolic genes were related to pre-operative muscle quality, and myogenic genes were related to pre-operative muscle size. These findings provide insight into molecular pathways associated with muscle health in the presence of lumbar spine pathology, establishing a foundation for future research that addresses how these changes impact outcomes in this patient population.

## Background

Lumbar spine pathology (LSP) is a common musculoskeletal condition leading to lower back and lower extremity pain and dysfunction. An estimated 75% of individuals with LSP experience suboptimal outcomes, including decreases in functional capacity of paraspinal muscles, ongoing pain and disability, recurrence of symptomatology often requiring surgical intervention, and a general lack of responsiveness to conservative interventions such as exercise-based rehabilitation [1–9]. Individuals with LSP typically demonstrate pathological changes in paraspinal musculature in comparison to healthy individuals [10–14]. The multifidus muscle is of particular interest due to its role in stabilizing the lumbar spine [15–17].

**Fig. 1:**
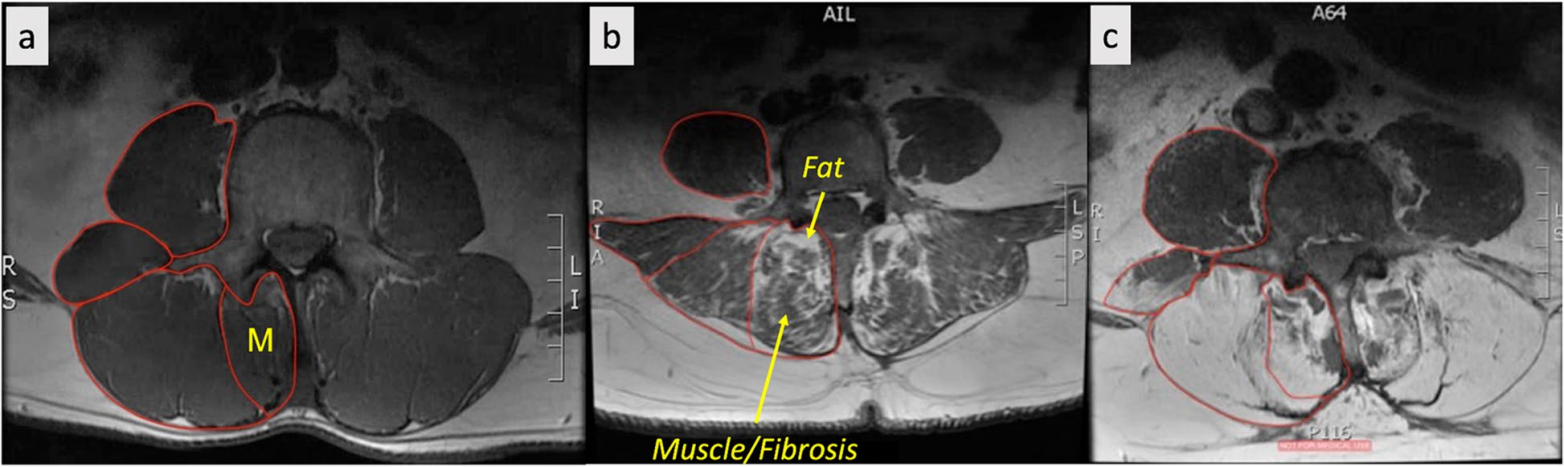
Pathological muscle adaptations. T2-weighted axial MRIs of the lumbar paraspinal muscles (red outlines) from three different individuals, depicting muscle degeneration. Panel (**a**) depicts an individual with minimal fatty infiltration of the multifidus (M) muscle compartment. Panel (**b**) depicts an individual with moderate fatty infiltration, while panel (**c**) depicts an individual with high fatty infiltration. In conventional T2-weighted MRI imaging, muscle and fibrosis both appear dark and fatty tissue appears bright, as depicted above

Commonly observed multifidus adaptations in individuals with LSP include increased accumulation of fat (Fig. 1), and collagen within muscle [10, 12, 13, 18–21]. Muscle atrophy has also been observed in some studies [13, 21–27]. Histological studies have similarly demonstrated muscle fiber degeneration [10, 19, 28], deposition of fibrotic tissues [10, 29, 30], increased inflammatory biomarkers [10, 31], and reductions in vascularity [10, 32] in paraspinal muscle biopsies from both humans and animals. Importantly, although paraspinal muscle degeneration has not been identified as a causal source for LSP, it has been associated with increased likelihood of symptom recurrence, poor post-operative outcomes, and reduced strength, endurance, and function, all of which may contribute to a poor prognosis in this patient population [7, 33–35]. The clinical implications of muscle health in the context of spine surgery is further highlighted by recent studies demonstrating a higher risk of proximal junctional kyphosis in patients with decreased back muscle volume and cross sectional area [36, 37]. Multifidus muscle degeneration was also associated with interbody cage subsidence in retrospective cohorts of patients undergoing both oblique and transforaminal lateral interbody fusions for LSP [38, 39].

Although the various studies referenced above reveal increasing knowledge surrounding the clinical relevance of paraspinal muscle health on surgical outcomes, few human studies exist describing the molecular mechanisms underlying pathological muscle in LSP [29, 40–43]. Prior work has demonstrated relationships between individual genes and paraspinal muscle degeneration [31, 44–47], which oversimplifies the underlying mechanisms behind muscle adaptation. To our knowledge, only one prior study has cumulatively looked at gene expression in the multiple pathways associated with muscle degeneration in individuals with LSP [29]. While this study compared gene expression in chronic versus acute LSP, it did not look at the association between gene expression levels and objective measures of muscle health. Muscle health and function is influenced by structural features such as size (cross-sectional area) and tissue composition (e.g., amount of fatty infiltration within the muscle compartment). For example, smaller muscles with higher levels of fatty infiltration demonstrate impaired performance and adaptation potential [48, 49]. However, literature on muscle-related impairments in the presence of LSP is lacking with respect to mechanism for impaired recovery potential. Therefore, the aim of this study is to investigate the relationship between macroscopic features of multifidus muscle pathology (atrophy and fatty infiltration) and transcriptional profiles representing impaired muscle recovery in individuals undergoing surgery for LSP. We hypothesized that (1) preoperative multifidus muscle size would be related to myogenic, atrophic, and fibrogenic gene expression and that (2) pre-operative multifidus fatty infiltration would be related to adipogenic gene expression. Understanding the tissue level adaptations in the presence of pathology will provide a springboard for identifying the most successful interventional approaches. Similarly, identifying defects in muscle recovery potential will provide insight into which combinations of treatments will be most likely to resolve structural and functional impairments in diseased muscle.”

## Methods

### Cohort

This was a cross sectional observational study of 59 individuals undergoing lumbar spine surgery for degenerative LSP. All patients consented to intraoperative biopsies of the multifidus muscle and were included if they were undergoing primary surgery including laminoforaminotomies, laminectomies, discectomies, or fusions (1 to 2 levels). Patients with any diagnosed myopathy or systemic neurological condition were excluded. This study was performed in accordance with the Declaration of Helsinki and under approval of the UC San Diego Institutional Review Board (IRB111647). Demographic and condition-specific characteristics including age, gender, symptom duration, diagnosis, Oswestry disability index (ODI), and Numerical pain rating scale (NPRS) rating were collected pre-operatively.

### Muscle Biopsy

Multifidus muscle biopsies were obtained intraoperatively during a posterior approach to the spine (*n*= 59). Muscle tissue was collected at a standardized anatomical location 1 cm lateral to the spinous process at the spinolaminar border at the level and side of primary pathology as previously described [10]. For patients with bilateral symptomatology, biopsies were obtained on the side of surgeon’s approach preference. Biopsies were handled using sterile techniques and immediately pinned at in-vivo length longitudinally using non-ferrous magnetic pins and cork, and flash frozen using liquid nitrogen-cooled isopentane. Biopsies were then transported on dry ice to the laboratory where they were stored at –80 °C until processing, as previously described [10, 29].

### RNA Isolation and Quantitative PCR

Approximately 25-50 mg of a given muscle biopsy was homogenized in a round bottom bead tube (Navy, NextAdvance) with 1 ml of QIAzol (Qiagen). RNeasy spin columns (Qiagen) were used to extract ribonucleic acid (RNA) by following the manufacturer’s protocol. Extracted RNA was analyzed for concentration and quality using QIAxpert Analysis (Qiagen). After determining acceptable purity and concentration, one microgram of complimentary deoxynucleic acid (cDNA) was reverse transcribed using the iScript cDNA Synthesis Kits (Biorad). Quantitative polymerase chain reaction (qPCR) was performed on custom plates (Biorad) on a BioRad CFX384 Touch qPCR analyzer for a panel of 42 genes associated with adipogenic/metabolic, atrophic, fibrogenic, inflammatory, and myogenic pathways, and 40S Ribosomal Protein (RPS18) and Beta-Actin (ACTB) as controls (Table 1).

**Table 1:** Genes and gene categories. Panel of 42 functional genes associated with adipogenic/metabolic, atrophic, fibrogenic, inflammatory, and myogenic pathways in skeletal muscle. Genes were examined using qPCR on custom cDNA plates containing these 42 genes of interest, and 40S Ribosomal Protein (RPS18) and Beta-Actin (ACTB) as controls

Gene Category	Adipogenic/ Metabolic	Atrophic	Fibrogenic	Inflammatory	Myogenic
Gene Name (Abbreviation)	Peroxisome Proliferator-Activated Receptor Gamma **(PPARG),** PPARG Coactivator 1 Alpha **(PPARGC1A),** Peroxisome Proliferator-Activated Receptor Delta **(PPARD),** Fatty Acid Binding Protein 4 (adipocyte specific) **(FABP4),** CCATT/Enhancer Binding Protein Alpha **(CEBPA),** Adiponectin **(ADIPOQ),** Wnt Family Member 10B **(WNT10B)** Protein Tyrosine Phosphatase Non-receptor Type 4 **(PTPN4)**	Myostatin/ Growth Differentiation Factor 8 **(MSTN),** Activin Receptor 2B **(ACVR2B),** Tripartite Motif Containing 63/E3 Ubiquitin Ligase **(TRIM63),** Forkhead Box O3 (**FOXO3),** F-box only protein 32 **(FBXO32)** Caspase-3 **(CASP3),** Caspase-1 **(CASP1)**	Platelet-Derived Growth Factor Receptor Alpha **(PDGFRA),** Tissue Inhibitor of Metalloproteinase 3 **(TIMP3),** Tissue Inhibitor of Metalloproteinase 1 **(TIMP1),** Matrix Metalloproteinase 9 **(MMP9),** Matrix Metalloproteinase 3 **(MMP3),** Matrix Metalloproteinase 1 **(MMP1),** Lysyl Oxidase **(LOX),** Fibronectin 1 **(FN1),** Connective Tissue Growth Factor **(CTGF),** Collagen Type III Alpha 1 Chain **(COL3A1),** Collagen type I Alpha Chain **(COL1A1),** Transforming Growth Factor Beta 1 **(TGFB1)**	Tumor Necrosis Factor **(TNF),** Interleukin-6 **(IL6),** Interleukin-10 **(IL10),** Interleukin-1 Beta **(IL1B)**	Embryonic Myosin Heavy Chain **(MYH3),** Myosin Heavy Chain – Type 1 **(MYH1),** Insulin-like Growth Factor I **(IGF-1),** Cysteine and Glycine Rich Protein 3/ Muscle LIM Protein **(CSRP3),** Ankyrin Repeat and SOCS Box Containing 15 **(ASB15),** Ankyrin Repeat Domain 2-Stretch Responsive Muscle **(ANKRD2),** Paired Box 7 Transcription Factor (**PAX7),** Myogenin/Myogenic Factor (**MYOG),** Myogenic Differentiation 1/Myogenic Factor 3 (**MYOD1),** Myogenic Factor 5 **(MYF5),** Mammalian Target of Rapamycin (**MTOR)**

Cycle threshold values (Ct-values) were determined using a SYBR green fluorophore. On-plate quality assessment was performed to assess genomic DNA contamination and RNA quality.

### Tissue composition

Ten-micron sections of each tissue biopsy were obtained from OCT-embedded frozen samples using a Leica (CM3050S, Buffalo Grove, USA) cryostat. Hematoxylin and Eosin (H&E) and Gomori Trichrome stains were used to visualize gross muscle morphology and quantify tissue content [50]. ImageJ (http://imagej.nih.gov/ij) was used to automatically quantify the relative fractions of muscle, adipose, loose collagen, and dense collagen in Trichrome-stained biopsy [51]. Briefly, tissue type was determined by manual intensity thresholding of the red (muscle), green (loose collagen), and blue (dense collage) channels of whole-section RGB images, while adipose tissue was identified morphologically and traced [50, 52]. This methodology has been previously used and described in detail [10, 42].

### Image analysis

**Fig. 2:**
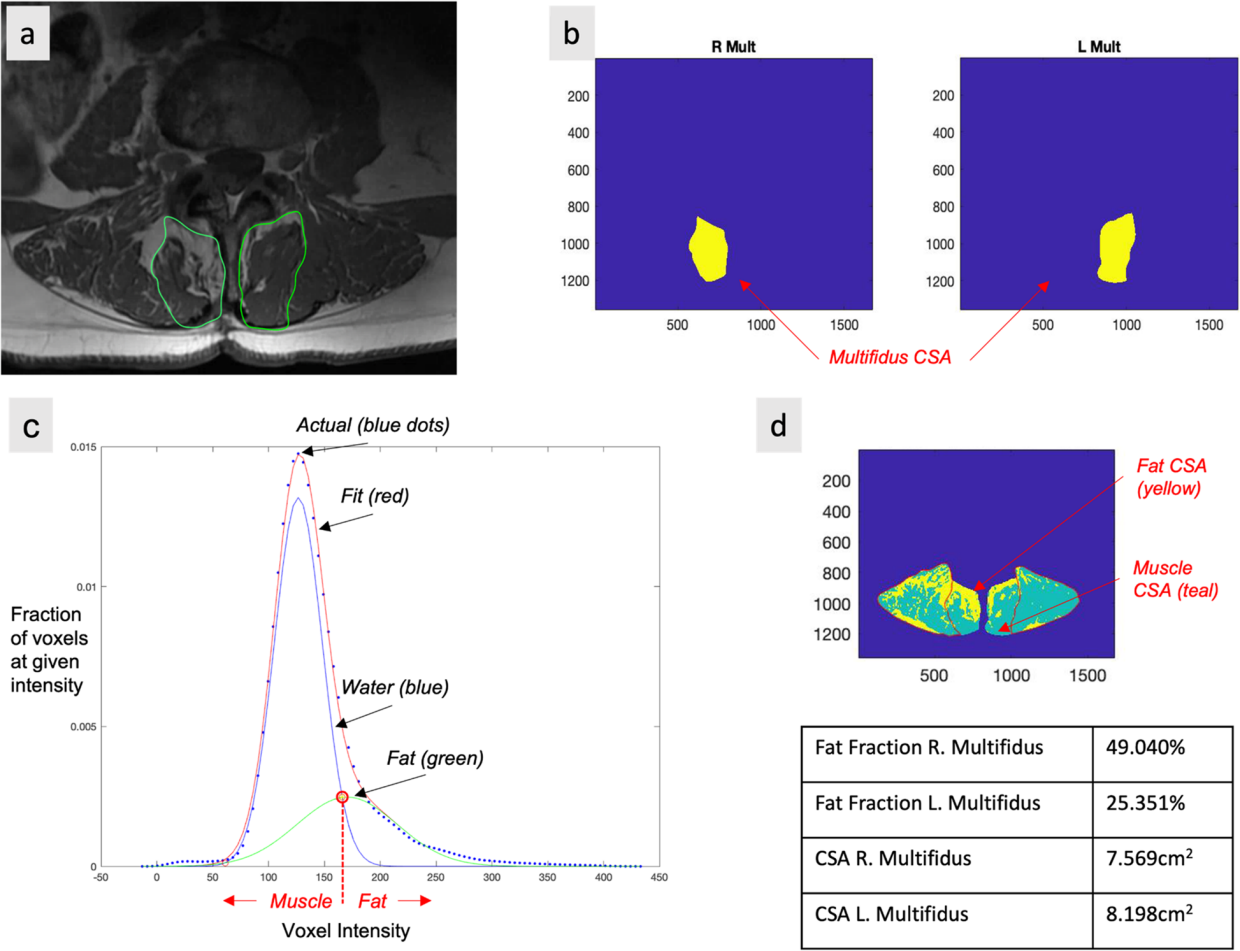
MRI Image Processing. Multifidus cross-sectional area and fat fraction were determined using custom MatLab code to differentiate between muscle and fat. Regions of interest were drawn around the right and left multifidus in T2-weighted axial MRIs of the lumbar spine (**a**). Right and left multifidus were evaluated for muscle size (**b**), then, a bi-gaussian distribution of voxel intensity (**c**) was used to determine the intercept (red circle) between water (blue line) and fat (green line). The intercept is used as a threshold; greater voxel intensities are designated as fat and lower voxel intensities are designated as muscle. This analysis highlights muscle as teal and fat as yellow (d) and provides a calculation for both right and left multifidus cross-sectional area and fat fraction (**d**, table)

Pre-operative T2-weighted clinical magnetic resonance images (MRIs) (*n* = 54) were obtained of the lumbar spine within the 6-months preceding surgery. All images were obtained using a 3 T MRI system (GE MR 750, GE Healthcare, Waukesha, WI, USA) and a spine array coil. Subjects were positioned head-first, supine and centered at the umbilicus. Axial multi-slice 2D images were obtained using the following imaging parameters: slice thickness = 8 mm with 0 gaps and 22 slices covering the entire lumbar spine from L1-S1, acquisition matrix = 128 × 128, FOV = 256 × 256 mm [2]. Open-source MRI processing software (Horos) was used to view and analyze axial MRI images. Muscle tissue was collected at a standardized anatomical location 1 cm lateral to the spinous process at the spinolaminar border at the level and side of primary pathology as previously described [10]. For patients with bilateral symptomatology, biopsies were obtained on the side of surgeon’s approach preference, and location level was verified using intraoperative fluoroscopy. Biopsy locations were matched to the MRI slice closest to the inferior vertebral endplate at the biopsy level for further analysis. Regions of interest (ROIs) were drawn to include the whole multifidus muscle compartment by a single rater who was present for the surgical procedure. The image assessor was a graduate student with training in image processing and spinal anatomy. These ROI methods have been previously described in detail and have been shown to have high inter- and intra-rater reliability with individuals of similar experience and training [53]. Images were analyzed for total multifidus compartment cross sectional area (CSA), lean muscle cross sectional area (M-CSA), fat cross sectional area (F-CSA), and proportion of fat within the muscle compartment (FF) using custom MatLab software (Fig. 2).

### Statistical analyses

Raw Ct-values were obtained from all samples (*n*= 59) and read into a qPCR expression set using the R Bioconductor package high-throughput qPCR (HTqPCR) [28]. Ct-values were then quantile normalized to the mean Ct-value to obtain gene expression values for each gene. Lower Ct values indicated higher gene expression. To allow for statistical comparison, a maximum Ct-value of 39 was applied to all genes of interest as this value approximated negligible gene expression [28]. Unsupervised hierarchical clustering using Euclidean distance was applied to normalized expression values and generate heat maps in order to visualize potential patterns of gene expression across patients.

Pearson correlations were performed between all genes and all MRI imaging measures using SPSS version 28.00 (IBM Corp. 2021) for all patients with available muscle biopsies and corresponding MRI imaging measures (*n* = 54). Raw p-values from correlations were adjusted for multiple comparisons within each gene group using the Benjamini & Hochberg method. Significance was set at an adjusted p-value threshold of *p* < 0.05, and trends were defined as an adjusted *p*-value < 0.1. Data are reported as mean with standard deviation (SD). If correlations were statistically significant, multivariate linear regression was used to adjust for potential confounding by age, gender, duration of symptoms, biopsy tissue composition, and diagnosis.

## Results

Of the 59 patients with muscle biopsies, 54 patients had matching preoperative MRI imaging (Table 2). Participants were on average, 51.5 (SD 16.9) years old, with moderate to severe pre-operative disability, averaging 44.9 (SD 21.1) points as measured by the ODI, and moderate pre-operative pain, averaging 5.6 (SD 2.8) points on the NPRS. Most participants were male (59%) and had chronic (> 3 months) symptoms (63%). Mean duration of symptoms was 29.5 months (SD 45.3). Histological tissue analysis demonstrated tissue samples to have a mean relative muscle fraction of 52.4% (SD 21.8). Diagnoses included disc herniation (*n* = 25), facet arthropathy (*n* = 15), degenerative spondylolisthesis (*n* = 11), and scoliosis or kyphoscoliosis (*n *= 3). The most common level of muscle biopsy was L4 (43%), followed by L3 (30%).

**Table 2:** Patient demographics. Reported age, ODI, NPRS, gender, duration of symptomatology, and diagnoses. Most patients underwent surgery for disc herniation

Patient Demographics	(*n* = 54)
Age (Years)—mean (SD)	51.5 (16.9)
ODI Baseline—mean (SD)	44.9 (21.1)
NPRS Baseline—mean (SD)	5.6 (2.8)
Gender (n)	
* Female*	22 (40.7%)
* Male*	32 (59.3%)
Duration of Symptomatology—mean (SD)	
* Mean length of symptoms in months*	29.5 (45.3)
Diagnosis (# of patients)	
* Disc Herniation*	25 (46.3%)
* Facet Arthropathy*	15 (27.7%)
* Degenerative Spondylolisthesis*	11 (20.4%)
* Scoliosis/kyphoscoliosis*	3 (5.5%)
Pathological Vertebral Level (# of patients)	
* L1*	1 (1.9%)
* L2*	0 (0%)
* L3*	16 (29.6%)
* L4*	23 (42.6%)
* L5*	14 (25.9%)

**Fig. 3:**
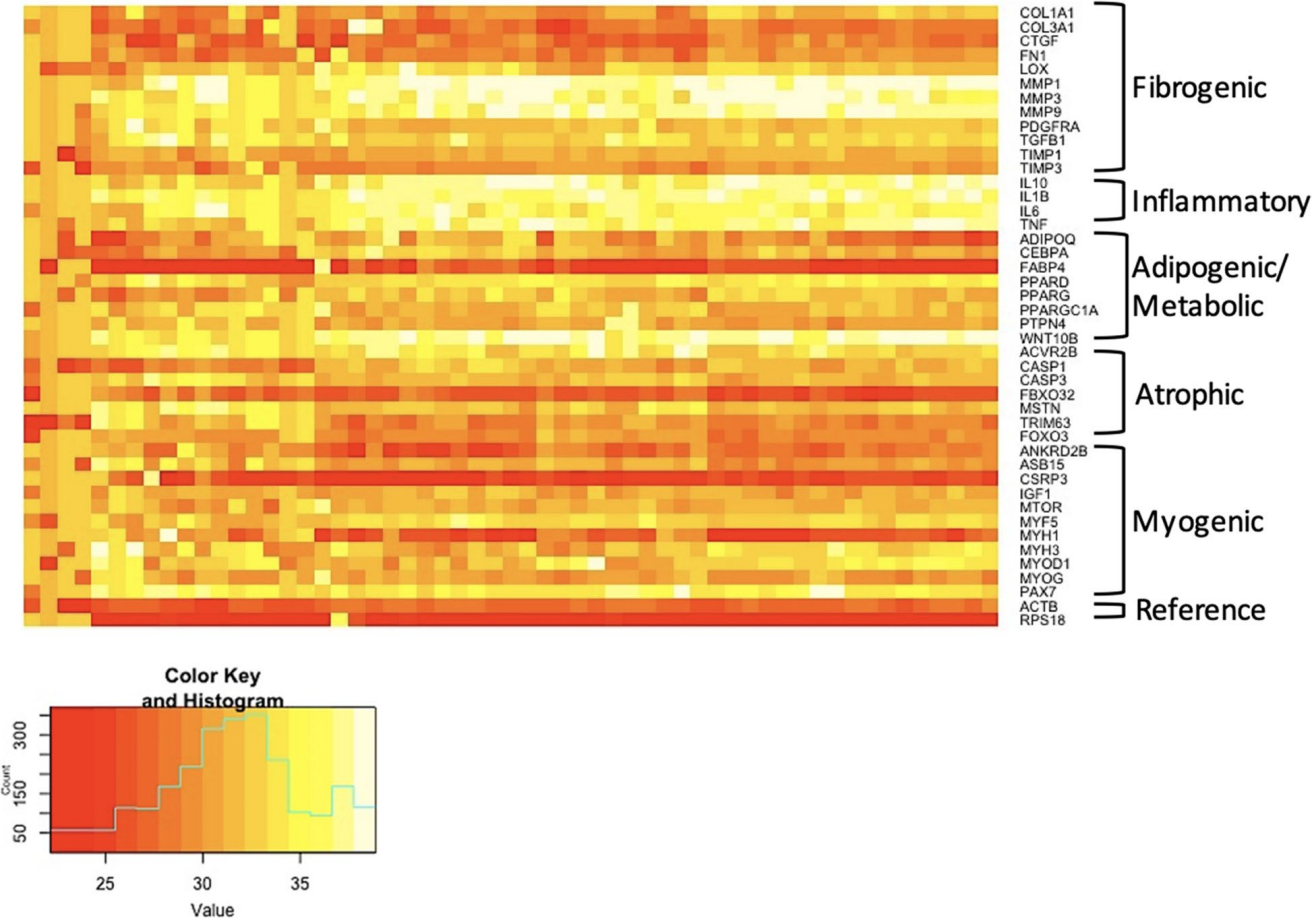
Gene heatmap of individuals undergoing surgery for lumbar spine pathology. Hierarchical cluster analysis of quantile normalized gene expression values from intraoperative multifidus muscle biopsies (*n* = 59). The expression levels of 42 genes from fibrogenic, inflammatory, adipogenic/metabolic, atrophic, and myogenic pathways were measured using qPCR. Gene abbreviations are indicated on the y-axis clustered by group (i.e., fibrogenic). Highly expressed genes are denoted by red coloring, and genes with low expression are denoted by yellow coloring

Normalized gene expression heat maps by gene category for all patients (*n* = 59) suggest higher expression of pro-fibrogenic genes, and lower expression of anti-fibrogenic and inflammatory genes in most participants (Fig. 3).

**Fig. 4:**
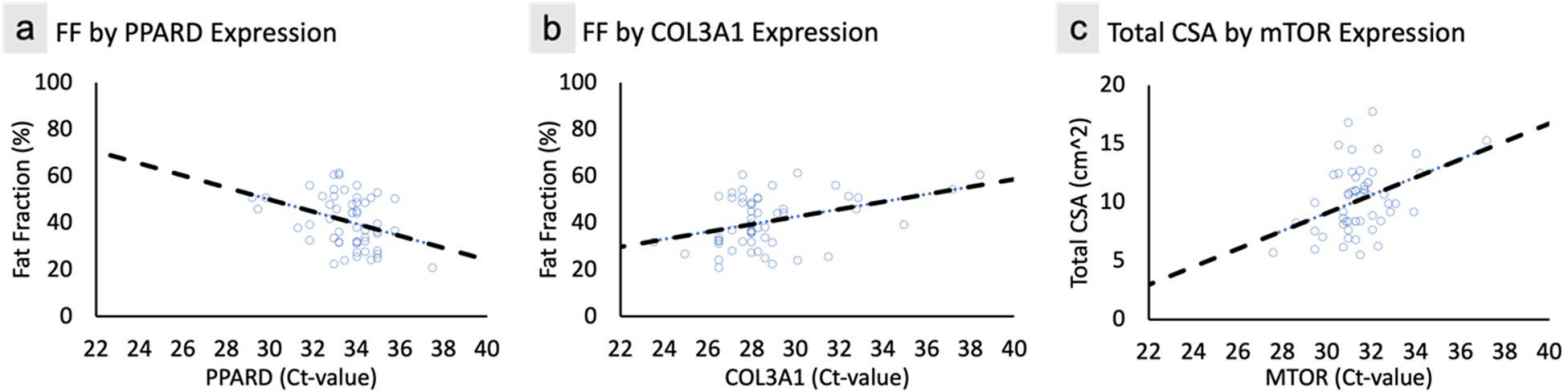
Associations between multifidus gene expression and pre-operative morphological measures. Raw Ct-values were quantile normalized to the mean Ct-value; lower Ct-values representing higher gene expression and vice versa. Individuals with greater fat fraction demonstrated (**a**) Increased expression of adipogenic/metabolic gene PPARD (*p* = 0.018, *r* = -0.346) and (**b**) decreased expression of fibrogenic gene COL3A1 (*p* = 0.047, *r* = 0.386). Individuals with lower multifidus cross-sectional area demonstrated (**c**) decreased expression of myogenic gene mTOR (*p* = 0.045, *r* = 0.388). Multifidus muscle biopsies were collected intra-operatively (*n* = 59) and gene expression was assessed using qPCR. Morphological measures were evaluated using pre-operative T2-weighted MRI of the lumbar spine at the level of multifidus biopsy (*n* = 54). Univariate analyses were performed between each gene and MRI measure, and p-values were adjusted for multiple comparison using the Benjamini & Hochberg method with an adjusted *p*-value threshold of *p* < 0.05, and trends were defined as adjusted *p*-values < 0.1

Relationships between gene expression and MRI (*n* = 54) revealed that lower expression of the adipogenic/metabolic gene PPARD (Fig. 4a), and higher expression of the fibrogenic gene COL3A1 (Fig. 4b), was associated with lower FF (*r* = -0.346, *p* = 0.018, and *r* = 0.386, *p* = 0.047 respectively). These relationships remained significant after correcting for diagnosis, gender, duration of symptoms, and relative fraction of muscle tissue, and were reduced to trends after correcting for age (*r* = -0.246, *p* = 0.076 for PPARD; and *r* = 0.26, *p* = 0.060 for COL3A1). For PPARD, the addition of age accounted for an increase in model variance (R^2^) explained from 23 to 36%. For every 1-year increase in age, an increase in fatty infiltration of 0.3% (SE 0.10%) is observed, *p* = 0.003. For COL3A1, the addition of age accounted for an increase in model variance explained from 26 to 37%. A similar beta weight for age was observed in this model (0.3%, SE 0.10%, *p* = 0.005). Additionally, lower expression of the myogenic gene mTOR (Fig. 4c) was related to greater total multifidus compartment cross sectional area (*r* = 0.388, *p* = 0.045). This relationship remained significant after correcting for diagnosis, gender, duration of symptoms, relative fraction of muscle tissue, and age.

## Discussion

The broad aim of this study was to investigate the relationship between expression patterns of genes relevant to muscle adaptation and paraspinal muscle health (size and composition) before spine surgery for LSP. Our observations supplement previous literature demonstrating an LSP phenotype in human and animal models consistent with increases in fatty tissue [7, 8, 10, 11, 18, 20, 23, 27, 30, 35, 54, 55], and increases in fibrotic tissue [7, 29, 30, 42, 54, 56, 57] within multifidus and paraspinal muscles. Additionally, our gene findings supplement prior literature describing upregulation of genes and gene pathways involved in skeletal uscle atrophy [58–63], fibrotic tissue deposition [29, 30, 42, 64–68], and fatty infiltration [30, 31] in the context of a degenerative muscle phenotype. Furthermore, our findings supplement the few studies that have looked at gene findings and muscle morphology in humans with LSP [29, 42]. The relationship between muscle health in LSP and genes involved in changes in muscle composition (including fibrosis and adipose tissue deposition) provides insight into the mechanisms underlying muscle adaptation, warranting investigation of possible relationships between objective health measures and such gene findings.

We found that individuals with higher levels of preoperative multifidus fatty infiltration demonstrated increased expression of adipogenic/metabolic genes, although this relationship covaried with age. PPARD is thought to trigger a switch from glucose/fatty acid metabolism to fatty acid metabolism, leading to reductions in body fat, although whether this is true in skeletal muscle is unknown [69]. The increased expression of PPARD may suggest that higher levels of intramuscular fat are driving greater metabolic demand.

We also observed that individuals with higher levels of multifidus fatty infiltration demonstrated lower expression of fibrogenic gene COL3A1. COL3A1 is one of various collagen isoforms that composes the extracellular matrix (ECM), secreted by muscle fibroblasts [70] in response to muscle injury [10, 29, 71]. Prior studies have shown upregulation of the COL1A1 isoform in humans with chronic lumbar pathology [29], however no studies have demonstrated significant associations with lower fat fraction in muscular phenotypes as seen in this study.

Our results demonstrated that age was more strongly associated with fat fraction than PPARD and COL3A1 expression, which is not surprising given that aging has been previously shown to be associated with greater multifidus fatty infiltration in individuals with LSP compared to healthy aging adults [11, 55, 72]. However, retention of a relationship between these genes and FF even after correcting for age may suggest that these genes are of interest for future investigations of muscle degeneration.

Regarding muscle composition and our MRI parameters, T2 imaging possesses limitations distinguishing between fat and inflammation, and between muscle and fibrosis. Other MRI methodologies such as fat–water separation techniques, 2-point DIXON, and IDEAL imaging may provide more accurate measures of fat content within the multifidus muscle compartment [73]. Additionally, muscle and fibrosis possess similar relaxometry times with short signal decay such that they both present as dark grey on MRI and are ultimately interpreted collectively as muscle using our current technique, which may result in an overestimation of muscle tissue and underestimation of fibrotic tissue within the multifidus muscle compartment [74]. Future studies using methods such as ultra-short echo-time (UTE) MRI may be able to differentiate between muscle and fibrosis due to short image acquisition times, which could help clarify the directionality and significance of these findings [74].

Finally, contrary to our hypothesis, we observed that individuals with larger multifidus CSA demonstrated decreased expression of pro-myogenic gene mTOR. Limitations in MRI acquisition technique may also help to explain our observation of decreased pro-myogenic gene expression of mTOR in individuals with greater multifidus muscle size. We expected to see increased expression of mTOR alongside greater multifidus muscle size, as mTOR is known to be a potent regulator of myogenesis [30].

This study has several limitations apart from those related to MRI acquisition. The gene expression patterns are cross sectional and thus provide no temporal resolution of the cellular mechanisms that may influence muscle changes over time. Additionally, skeletal muscle transcriptome patterns may be influenced by increasing age [75], which is not well understood in either a healthy or pathological context and may influence and confound gene expression patterns across subjects of different ages. Future studies in which fractional synthetic rates and protein abundance are measured may be more informative at the time of surgery given the chronic nature of LSP. Furthermore, while there were statistically significant associations between gene expression and muscle health as measured on MRI, we do acknowledge that the strength of these associations does not imply physiologic or clinical significance, especially without a comparison of normal healthy paraspinal tissue for reference. While future research would ideally include a healthy comparison to understand the significance of these findings, obtaining such tissue is often met with significant ethical and operational barriers. Finally, future research may look at how gene expression patterns relate longitudinally to post-operative muscle health.

## Conclusion

This study evaluated the relationship between multiple gene pathways and parameters of muscle health in humans with LSP. Although our preliminary findings suggest that some relationships between adipogenic, myogenic, and fibrogenic genes and muscle health phenotypes exist in individuals with LSP, these findings should be interpreted with caution as our findings were only in a small number of genes and our muscle health metrics were limited by our MRI methodology. Importantly, these relationships should be acknowledged in the context of patient age. These findings add to the growing knowledge on the molecular underpinnings of muscle degeneration in human lumbar musculoskeletal disease and can help direct future research to further delineate relationships between gene expression and muscle health. Continued clarification regarding these molecular mechanisms can provide important potential targets for future research, with the end goal of improving patient function, recovery, and prognosis.

## Data Availability

The datasets used and/or analyzed during the current study are available from the corresponding author on reasonable request.

## Abbreviations

CSA: Compartment cross sectional area
cDNA: Complimentary deoxynucleic acid
ECM: Extracellular matrix
F-CSA: Fat cross sectional area
FF: Proportion of fat within the muscle compartment
LSP: Lumbar spine pathology
M-CSA: Lean muscle cross sectional area
MRI: Magnetic resonance imaging
NPRS: Numerical pain rating scale
ODI: Oswestry disability index
qPCR: Quantitative polymerase chain reaction
SD: Standard deviation
UTE: Ultra-short echo-time

## References

[1] Cassidy JD, Côté P, Carroll LJ (2005). Incidence and course of low back pain episodes in the general population. Spine (Phila Pa 1976).

[2] Hestbaek L, Leboeuf-Yde C, Manniche C (2003). Low back pain: what is the long-term course? A review of studies of general patient populations. Eur Spine J.

[3] Verkerk K, Luijsterburg PAJ, Heymans MW (2013). Prognosis and course of disability in patients with chronic nonspecific low back pain: a 5- and 12-month follow-up cohort study. Phys Ther.

[4] Carey TS, Garrett JM, Jackman A (1999). Recurrence and care seeking after acute back pain: results of a long-term follow-up study North Carolina Back Pain Project. Med Care..

[5] Andersson GB (1999). Epidemiological features of chronic low-back pain. The Lancet.

[6] Rundell SD, Sherman KJ, Heagerty PJ (2015). The clinical course of pain and function in older adults with a new primary care visit for back pain. J Am Geriatr Soc.

[7] Airaksinen O, Herno A, Kaukanen E (1996). Density of lumbar muscles 4 years after decompressive spinal surgery. Eur Spine J.

[8] Storheim K, Berg L, Hellum C (2017). Fat in the lumbar multifidus muscles - predictive value and change following disc prosthesis surgery and multidisciplinary rehabilitation in patients with chronic low back pain and degenerative disc: 2-year follow-up of a randomized trial. BMC Musculoskelet Disord.

[9] Steele J, Fisher J, Bruce-Low S (2017). Variability in Strength, Pain, and Disability Changes in Response to an Isolated Lumbar Extension Resistance Training Intervention in Participants with Chronic Low Back Pain. Healthcare.

[10] Shahidi B, Hubbard JC, Gibbons MC (2017). Lumbar multifidus muscle degenerates in individuals with chronic degenerative lumbar spine pathology: Multifidus Degeneration in Lower Back Pain. J Orthop Res.

[11] Shahidi B, Parra CL, Berry DB (2017). Contribution of Lumbar Spine Pathology and Age to Paraspinal Muscle Size and Fatty Infiltration. Spine.

[12] Hodges PW, Danneels L (2019). Changes in Structure and Function of the Back Muscles in Low Back Pain: Different Time Points, Observations, and Mechanisms. J Orthop Sports Phys Ther.

[13] Barker KL, Shamley DR, Jackson D (2004). Changes in the cross-sectional area of multifidus and psoas in patients with unilateral back pain: the relationship to pain and disability. Spine (Phila Pa 1976).

[14] Demoulin C, Crielaard J-M, Vanderthommen M (2007). Spinal muscle evaluation in healthy individuals and low-back-pain patients: a literature review. Joint Bone Spine.

[15] Brown SHM, Banuelos K, Ward SR (2010). Architectural and morphological assessment of rat abdominal wall muscles: comparison for use as a human model: Rat abdominal muscle architecture. J Anat.

[16] Regev GJ, Kim CW, Tomiya A (2011). Psoas Muscle Architectural Design, In Vivo Sarcomere Length Range, and Passive Tensile Properties Support Its Role as a Lumbar Spine Stabilizer. Spine.

[17] Ward SR, Kim CW, Eng CM (2009). Architectural analysis and intraoperative measurements demonstrate the unique design of the multifidus muscle for lumbar spine stability. J Bone Joint Surg Am.

[18] Alaranta H, Tallroth K, Soukka A (1993). Fat content of lumbar extensor muscles and low back disability: a radiographic and clinical comparison. J Spinal Disord.

[19] Ng JK, Richardson CA, Kippers V (1998). Relationship between muscle fiber composition and functional capacity of back muscles in healthy subjects and patients with back pain. J Orthop Sports Phys Ther.

[20] Wan Q, Lin C, Li X (2015). MRI assessment of paraspinal muscles in patients with acute and chronic unilateral low back pain. Br J Radiol.

[21] Danneels LA, Vanderstraeten GG, Cambier DC (2000). CT imaging of trunk muscles in chronic low back pain patients and healthy control subjects. Eur Spine J.

[22] Kamaz M, Kireşi D, Emlik D (2007). CT measurement of trunk muscle areas in patients with chronic low back pain. Diagn Interv Radiol.

[23] Kang CH, Shin MJ, Kim SM (2007). MRI of paraspinal muscles in lumbar degenerative kyphosis patients and control patients with chronic low back pain. Clin Radiol.

[24] Hides J, Gilmore C, Stanton W (2008). Multifidus size and symmetry among chronic LBP and healthy asymptomatic subjects. Man Ther.

[25] Wallwork TL, Stanton WR, Freke M (2009). The effect of chronic low back pain on size and contraction of the lumbar multifidus muscle. Man Ther.

[26] Kim WH, Lee SH, Lee DY (2011). Changes in the cross-sectional area of multifidus and psoas in unilateral sciatica caused by lumbar disc herniation. J Korean Neurosurg Soc.

[27] Chan ST, Fung PK, Ng NY (2012). Dynamic changes of elasticity, cross-sectional area, and fat infiltration of multifidus at different postures in men with chronic low back pain. Spine J.

[28] Mannion AF, Weber BR, Dvorak J (1997). Fibre type characteristics of the lumbar paraspinal muscles in normal healthy subjects and in patients with low back pain. J Orthop Res.

[29] Shahidi B, Fisch KM, Gibbons MC (2020). Increased Fibrogenic Gene Expression in Multifidus Muscles of Patients With Chronic Versus Acute Lumbar Spine Pathology. Spine.

[30] Hodges PW, James G, Blomster L (2015). Multifidus muscle changes after back injury are characterized by structural remodeling of muscle, adipose and connective tissue, but not muscle atrophy: molecular and morphological evidence. Spine.

[31] Hodges PW, James G, Blomster L (2014). Can Proinflammatory Cytokine Gene Expression Explain Multifidus Muscle Fiber Changes After an Intervertebral Disc Lesion?. Spine.

[32] Hiepe P, Gussew A, Rzanny R (2015). Age-related structural and functional changes of low back muscles. Exp Gerontol.

[33] Kaser L, Mannion AF, Rhyner A, et al. Active Therapy for Chronic Low Back Pain n.d.:11.

[34] Käser L, Mannion AF, Rhyner A (2001). Active Therapy For Chronic Low Back Pain: Part 2 Effects on Paraspinal Muscle Cross-Sectional Area, Fiber Type Size, and Distribution. Spine (Phila Pa 1976).

[35] D’hooge R, Cagnie B, Crombez G (2012). Increased intramuscular fatty infiltration without differences in lumbar muscle cross-sectional area during remission of unilateral recurrent low back pain. Man Ther.

[36] Yuan L, Zeng Y, Chen Z, Li W, Zhang X, Mai S (2021). Degenerative lumbar scoliosis patients with proximal junctional kyphosis have lower muscularity, fatty degeneration at the lumbar area. Eur Spine J.

[37] Kim DK, Kim JY, Kim DY, Rhim SC, Yoon SH (2017). Risk Factors of Proximal Junctional Kyphosis after Multilevel Fusion Surgery: More Than 2 Years Follow-Up Data. J Korean Neurosurg Soc.

[38] Kotheeranurak V, Jitpakdee K, Lin GX, Mahatthanatrakul A, Singhatanadgige W, Limthongkul W, Yingsakmongkol W, Kim JS (2021). Subsidence of interbody cage following oblique lateral interbody fusion: an analysis and potential risk factors. Global Spine J..

[39] Singhatanadgige W, Sukthuayat A, Tanaviriyachai T, Kongtharvonskul J, Tanasansomboon T, Kerr SJ, Limthongkul W (2021). Risk factors for polyetheretherketone cage subsidence following minimally invasive transforaminal lumbar interbody fusion. Acta Neurochir (Wien).

[40] Morris P, Ali K, Merritt M (2020). A systematic review of the role of inflammatory biomarkers in acute, subacute and chronic non-specific low back pain. BMC Musculoskelet Disord.

[41] Klyne DM, Barbe MF, Van Den Hoorn W (2018). ISSLS Prize in Clinical Science 2018: longitudinal analysis of inflammatory, psychological, and sleep-related factors following an acute low back pain episode—the good, the bad, and the ugly. Eur Spine J.

[42] Shahidi B, Gibbons MC, Esparza M, et al. Cell populations and muscle fiber morphology associated with acute and chronic muscle degeneration in lumbar spine pathology. JOR Spine 2020;3:. 10.1002/jsp2.1087. 10.1002/jsp2.1087 PMC7323470 32613162

[43] Attia E, Brown H, Henshaw R (2009). Patterns of Gene Expression in a Rabbit Partial Anterior Cruciate Ligament Transection Model: The Potential Role of Mechanical Forces. Am J Sports Med.

[44] Gibbons MC, Fisch KM, Pichika R (2018). Heterogeneous muscle gene expression patterns in patients with massive rotator cuff tears. PLoS ONE.

[45] Morales MG, Cabello-Verrugio C, Santander C (2011). CTGF/CCN-2 over-expression can directly induce features of skeletal muscle dystrophy. J Pathol.

[46] Sun G, Haginoya K, Chiba Y (2010). Elevated plasma levels of tissue inhibitors of metalloproteinase-1 and their overexpression in muscle in human and mouse muscular dystrophy. J Neurol Sci.

[47] Dvinge H, Bertone P (2009). HTqPCR: high-throughput analysis and visualization of quantitative real-time PCR data in R. Bioinformatics.

[48] Lieber RL, Ward SR (2011). Skeletal muscle design to meet functional demands. Philos Trans R Soc Lond B Biol Sci.

[49] Ward SR, Kim CW, Eng CM, Gottschalk LJ, Tomiya A, Garfin SR, Lieber RL (2009). Architectural analysis and intraoperative measurements demonstrate the unique design of the multifidus muscle for lumbar spine stability. J Bone Joint Surg Am.

[50] Miller JL, Watkin KL, Chen MF (2002). Muscle, adipose, and connective tissue variations in intrinsic musculature of the adult human tongue. J Speech Lang Hear Res.

[51] Abràmoff MD, Magalhães PJ, Ram SJ (2004). Image processing with imageJ. Biophoton Int.

[52] Gibbons MC, Singh A, Anakwenze O, Cheng T, Pomerantz M, Schenk S, Engler AJ, Ward SR (2017). Histological Evidence of Muscle Degeneration in Advanced Human Rotator Cuff Disease. J Bone Joint Surg Am.

[53] Berry DB, Padwal J, Johnson S, Parra CL, Ward SR, Shahidi B (2018). Methodological considerations in region of interest definitions for paraspinal muscles in axial MRIs of the lumbar spine. BMC Musculoskelet Disord.

[54] Brown SHM, Gregory DE, Carr JA (2011). ISSLS Prize Winner: Adaptations to the Multifidus Muscle in Response to Experimentally Induced Intervertebral Disc Degeneration. Spine.

[55] Fortin M, Videman T, Gibbons LE, Battié MC (2014). Paraspinal muscle morphology and composition: a 15-yr longitudinal magnetic resonance imaging study. Med Sci Sports Exerc.

[56] James G, Millecamps M, Stone LS (2018). Dysregulation of the Inflammatory Mediators in the Multifidus Muscle After Spontaneous Intervertebral Disc Degeneration SPARC-null Mice is Ameliorated by Physical Activity. Spine (Phila Pa 1976).

[57] James G, Klyne DM, Millecamps M (2019). ISSLS Prize in Basic science 2019: Physical activity attenuates fibrotic alterations to the multifidus muscle associated with intervertebral disc degeneration. Eur Spine J.

[58] Mammucari C, Milan G, Romanello V (2007). FoxO3 controls autophagy in skeletal muscle in vivo. Cell Metab.

[59] Fitzwalter BE, Thorburn A (2018). FOXO3 links autophagy to apoptosis. Autophagy.

[60] Sandri M, Sandri C, Gilbert A (2004). Foxo transcription factors induce the atrophy-related ubiquitin ligase atrogin-1 and cause skeletal muscle atrophy. Cell.

[61] Sandri M, Barberi L, Bijlsma AY (2013). Signalling pathways regulating muscle mass in ageing skeletal muscle: the role of the IGF1-Akt-mTOR-FoxO pathway. Biogerontology.

[62] Csibi A, Leibovitch MP, Cornille K (2009). MAFbx/Atrogin-1 controls the activity of the initiation factor eIF3-f in skeletal muscle atrophy by targeting multiple C-terminal lysines. J Biol Chem.

[63] Bodine SC, Baehr LM (2014). Skeletal muscle atrophy and the E3 ubiquitin ligases MuRF1 and MAFbx/atrogin-1. Am J Physiol Endocrinol Metab.

[64] Chen X, Li Y (2009). Role of matrix metalloproteinases in skeletal muscle: migration, differentiation, regeneration and fibrosis. Cell Adh Migr.

[65] Mackey AL, Brandstetter S, Schjerling P (2011). Sequenced response of extracellular matrix deadhesion and fibrotic regulators after muscle damage is involved in protection against future injury in human skeletal muscle. FASEB J.

[66] Morales MG, Acuña MJ, Cabrera D (2018). The pro-fibrotic connective tissue growth factor (CTGF/CCN2) correlates with the number of necrotic-regenerative foci in dystrophic muscle. J Cell Commun Signal.

[67] Liu H, Chen SE, Jin B (2010). TIMP3: a physiological regulator of adult myogenesis. J Cell Sci.

[68] Lee YS, Kim JY, Oh KS (2017). Fatty acid-binding protein 4 regulates fatty infiltration after rotator cuff tear by hypoxia-inducible factor 1 in mice. J Cachexia Sarcopenia Muscle.

[69] Riserus U, Sprecher D, Johnson T (2008). Activation of Peroxisome Proliferator-Activated Receptor (PPAR) Promotes Reversal of Multiple Metabolic Abnormalities, Reduces Oxidative Stress, and Increases Fatty Acid Oxidation in Moderately Obese Men. Diabetes.

[70] Sanderson RD, Fitch JM, Linsenmayer TR (1986). Fibroblasts promote the formation of a continuous basal lamina during myogenesis in vitro. J Cell Biol.

[71] Vafiadaki E, Arvanitis DA, Papalouka V (2014). Muscle lim protein isoform negatively regulates striated muscle actin dynamics and differentiation. FEBS J.

[72] Crawford RJ, Filli L, Elliott JM (2016). Age- and Level-Dependence of Fatty Infiltration in Lumbar Paravertebral Muscles of Healthy Volunteers. AJNR Am J Neuroradiol..

[73] Smith AC, Parrish TB, Abbott R, Hoggarth MA, Mendoza K, Chen YF, Elliott JM (2014). Muscle-fat MRI: 1.5 Tesla and 3.0 Tesla versus histology. Muscle Nerve.

[74] Schuijf JD, Ambale-Venkatesh B, Kassai Y (2019). Cardiovascular ultrashort echo time to map fibrosis—promises and challenges. BJR.

[75] Tumasian RA, Harish A, Kundu G, Yang JH, Ubaida-Mohien C, Gonzalez-Freire M, Kaileh M, Zukley LM, Chia CW, Lyashkov A, Wood WH, Piao Y, Coletta C, Ding J, Gorospe M, Sen R, De S, Ferrucci L (2021). Skeletal muscle transcriptome in healthy aging. Nat Commun.

[76] Rashid MM, Runci A, Russo MA (2015). Muscle Lim Protein (MLP)/CSRP3 at the crossroad between mechanotransduction and autophagy. Cell Death Dis.

[77] Vafiadaki E, Arvanitis DA, Sanoudou D (2015). Muscle LIM Protein: Master regulator of cardiac and skeletal muscle functions. Gene.

[78] Han S, Cui C, Wang Y (2019). Knockdown of CSRP3 inhibits differentiation of chicken satellite cells by promoting TGF-β/Smad3 signaling. Gene.

[79] Jensen JH, Conley LN, Hedegaard J (2012). Gene expression profiling of porcine skeletal muscle in the early recovery phase following acute physical activity. Exp Physiol.

[80] Li XZ, Yan Y, Zhang JF (2019). Oleic acid in the absence of a PPARγ agonist increases adipogenic gene expression in bovine muscle satellite cells1. J Anim Sci.

[81] Qiao L, Zou C, Shao P (2008). Transcriptional regulation of fatty acid translocase/CD36 expression by CCAAT/enhancer-binding protein alpha. J Biol Chem.

[82] Cavanaugh EJ, DiMario JX (2016). C/EBPα represses slow myosin heavy chain 2 gene expression in developing avian myotubes. Biochim Biophys Acta.

[83] Kjaer P, Bendix T, Sorensen JS (2007). Are MRI-defined fat infiltrations in the multifidus muscles associated with low back pain?. BMC Med.

[84] Albert HB, Briggs AM, Kent P (2011). The prevalence of MRI-defined spinal pathoanatomies and their association with modic changes in individuals seeking care for low back pain. Eur Spine J.

